# *Leishmania infantum* Parasites Subvert the Host Inflammatory Response through the Adenosine A2_A_ Receptor to Promote the Establishment of Infection

**DOI:** 10.3389/fimmu.2017.00815

**Published:** 2017-07-20

**Authors:** Mikhael H. F. Lima, Lais A. Sacramento, Gustavo F. S. Quirino, Marcela D. Ferreira, Luciana Benevides, Alynne K. M. Santana, Fernando Q. Cunha, Roque P. Almeida, João S. Silva, Vanessa Carregaro

**Affiliations:** ^1^Laboratory of Immunoparasitology, Department of Biochemistry and Immunology, School of Medicine of Ribeirão Preto, University of São Paulo, Ribeirão Preto, São Paulo, Brazil; ^2^Laboratory of Molecular Biology, Center for Biology and Health Sciences, Federal University of Sergipe, Aracaju, Sergipe, Brazil; ^3^Laboratory of Inflammation and Pain, Department of Pharmacology, School of Medicine of Ribeirão Preto, University of São Paulo, Ribeirão Preto, São Paulo, Brazil

**Keywords:** visceral leishmaniasis, adenosine, immunoregulation, A_2A_R signaling, inflammation

## Abstract

Adenosine is an endogenously released purine nucleoside that signals through four widely expressed G protein-coupled receptors: A1, A2_A_, A2_B_, and A3. Of these, A_2A_R is recognized as mediating major adenosine anti-inflammatory activity. During cutaneous leishmaniasis, adenosine induces immunosuppression, which promotes the establishment of infection. Herein, we demonstrated that A_2A_R signaling is exploited by *Leishmania infantum* parasites, the etiologic agent that causes Visceral Leishmaniasis, to successfully colonize the vertebrate host. A_2A_R gene-deleted mice exhibited a well-developed cellular reaction with a strong Th1 immune response in the parasitized organs. An intense infiltration of activated neutrophils into the disease-target organs was observed in A_2A_R^−/−^ mice. These cells were characterized by high expression of CXCR2 and CD69 on their cell surfaces and increased *cxcl1* expression. Interestingly, this phenotype was mediated by IFN-γ on the basis that a neutralizing antibody specific to this cytokine prevented neutrophilic influx into parasitized organs. In evaluating the immunosuppressive effects, we identified a decreased number of CD4^+^ FOXP3^+^ T cells and reduced *il10* expression in A_2A_R^−/−^ infected mice. During *ex vivo* cell culture, A_2A_R^−/−^ splenocytes produced smaller amounts of IL-10. In conclusion, we demonstrated that the A_2A_R signaling pathway is detrimental to development of Th1-type adaptive immunity and that this pathway could be associated with the regulatory process. In particular, it promotes parasite surveillance.

## Introduction

*Leishmania* parasites are the etiological agent of a wide spectrum of diseases in mammals and other vertebrates (1). Among this complex of diseases, Visceral Leishmaniasis (VL), which is caused by *L. donovani* or *L. infantum*, is one of the most severe clinical manifestations of infection with *Leishmania* parasites and is a major cause of human mortality and morbidity worldwide (2–4).

The most effective mechanisms for protection against *Leishmania* involve the generation of CD4^+^ Th1 cells. These cells secrete IFN-γ, which activates phagocytic cells, such as neutrophils, macrophages, and dendritic cells (DCs), to release reactive oxygen species (ROS) and nitric oxide (NO). These mediators lead to the death of the parasites (5, 6). IL-17, which is produced by the Th17 subset, can act synergistically with IFN-γ to increase the NO production and the anti-Leishmanial ability of the infected macrophages (7). Despite having several microbicidal activities to control parasite growth, the host defense can be subverted by the *Leishmania* parasite to provide a typical microenvironment for initiation and maintenance of successful infection. The mechanisms that are altered could involve those mediated by cellular response [i.e., Th2 subset, regulatory T cells (Tregs)], anti-inflammatory cytokines (IL-10, TGF-β), and some metabolites that have a high capacity to inhibit leukocyte migration and activation (8), including arachidonic acid metabolites (Prostaglandins E and J series) and adenosine (9, 10).

Adenosine is a potent immunomodulatory biomolecule that is produced by the ecto-enzymes CD39 (nucleoside triphosphate dephosphorylase) and CD73 (ecto-5′-nucleotidase), which are highly expressed by several cell types including leukocytes during stress, injury, and infection (11). Under these circumstances, extracellular ATP is hydrolyzed by CD39, which converts ATP or ADP into AMP, and subsequently CD73 rapidly dephosphorylates AMP to adenosine (ADO) (12, 13). After being generated, adenosine modulates the immunological responses through the activation of four G-protein-coupled transmembrane receptors (GPCRs) that can either stimulate (Gs) or inhibit (Gi) adenylyl cyclase, which catalyzes the formation of cyclic AMP (cAMP), which inhibits immune cell function. The adenosine A1 and A3 receptors are high- and low-affinity receptors for adenosine, respectively, and both are coupled to Gi, which decreases the generation of cAMP. By contrast, the high-affinity A2_A_ and low-affinity A2_B_ receptors activate adenylyl cyclase, thereby increasing the intracellular levels of cAMP (14, 15). Thus, A_2A_R and A_2B_R regulate multiple physiologic responses, including the anti-inflammatory and immunosuppressive effects of ADO. Genetic ablation or pharmacologic inhibition of A_2A_R or A_2B_R leads to excessive immune responses (16, 17).

The A_2A_R is widely distributed on the surfaces of several types of leukocytes, including neutrophils, monocytes, macrophages, DCs, T cells, and natural killer (NK) cells (18). Among its activities, A_2A_R activation blocks the classical macrophage activation by inhibiting its microbicidal machinery (19), attenuating phagocytosis (20), and blocking the production of ROS by phagocytes (21, 22). Moreover, A_2A_R signaling reduces the leukocyte recruitment to inflammatory foci (23, 24), induces T cell anergy (25, 26), and promotes both regulatory T cell generation and suppressive functions (27, 28). Furthermore, adenosine, acting through A_2A_R signaling, inhibits the DC ability to present antigen, thus leading to suppression of the Th17 subset dependent on IL-10 production (29).

In addition to these effects on the host cells, there is increasing evidence that microorganisms escape from the control of the immune system due to the synthesis of adenosine at the site of infection, which favors invasion and dissemination of infectious agents. Several microorganisms, including protozoa (belonging to the genus *Trypanosoma, Toxoplasma, Trichomonas, Giardia*) (30–36), fungi (*Candida parapsilosis*) (37), bacteria (38–40), and worms (*Schistosoma mansoni* and *S. japonicum*) (41, 42) express CD39–CD73-like machinery that may aid pathogen colonization and dissemination. *Leishmania* parasites can also take advantage of ectonucleotidases expressed on their membrane surfaces to escape from immunological surveillance (32, 43–45). In cutaneous leishmaniasis, we previously demonstrated that ADO and AMP present in saliva of *Phlebotomus papatasi, a Leishmania vector*, mediate the immunosuppressive effects. ADO and AMP act through A_2A_R to induce a tolerogenic profile on dendritic cells by sequential production of PGE_2_ and IL-10. Both mediators could also act in a paracrine manner to induce Tregs from Teff populations, thus leading to suppression of the immune response and parasite spreading (46).

An interesting issue is that, in general, the visceral *Leishmania* species are less inflammatory than the cutaneous *Leishmania* species (47). Intriguingly, the viscerotropic *Leishmania* species (*L. infantum* and *L. donovani*) demonstrate higher 3′-nucleotidase activity than the cutaneous species (48). Furthermore, patients with VL have high levels of adenosine in their serum, which is related to the ectonucleotidase activities and disease progression (49). In addition, under inflammatory conditions, A_2B_R is highly expressed in the monocytes from VL patients (50), which suggests that during this disease, the *Leishmania* parasites may use the adenosinergic signaling pathway to evade host immune response, which contributes to their silent growth and survival inside cells. However, the role of high-affinity A_2A_R receptor on VL remains to be elucidated. In this context, in this study, we demonstrate that the *L. infantum* parasite benefits from purinergic signaling mediated by the A_2A_R pathway in the host cells to subvert the immune response. Mechanistically, A_2A_R signaling negatively regulates the migration and activation of neutrophils that are induced by Th1 cells, thus allowing the establishment of the infection caused by parasite in the susceptible BALB/c mice.

## Materials and Methods

### Mice

Female BALB/c (wild type; WT) and BALB/c-A_2A_R^−/−^ (A_2A_R^−/−^) mice that weighed between 18 and 22 g were housed in the animal facility of the Department of Biochemistry and Immunology, School of Medicine of Ribeirão Preto, University of São Paulo (Brazil) in temperature-controlled rooms (22–25°C) and received water and food *ad libitum*. All experiments were conducted in accordance with the National Institutes of Health (NIH) guidelines on the welfare of experimental animals and with the approval of the Ethics Committee of the School of Medicine of Ribeirão Preto (No 196/2011).

### Parasites, Infection, and Parasites Load Estimation

Isolate HU-UFS14 of *L. infantum* was cultured in Schneider medium supplemented with 20% heat-inactivated fetal bovine serum (Gibco^®^, Life Technologies, Carlsbad, CA, USA), 5% penicillin and streptomycin (all from Sigma-Aldrich, St. Louis, MO, USA), and 2% human male urine. The mice were intravenously infected with 10^7^ of the promastigote form of *L. infantum* parasites in the stationary growth phase. The hepatic and splenic parasite burdens were determined using a quantitative limiting dilution assay (51, 52).

### Histopathological and Immunohistochemical Analyses

The mice were euthanized 0, 4, and 6 weeks after infection, and their livers were removed. The tissues were fixed in formalin, dehydrated in graded ethanol, and embedded in paraffin. Serial sections (5 µm) were cut and mounted on glass slides that had been precoated with 0.1% poly-l-lysine (Sigma-Aldrich). Histological assessment was performed after routine hematoxylin-eosin staining. The extent of granuloma formation was analyzed in 50 fields per animal, being classified as: none granuloma, which is characterized by some parasitized cells but in the absence of inflammatory cells surrounding them; developing granuloma, which is characterized as parasitized cells surrounded by some inflammatory leukocytes; mature granuloma, in which the fused parasitized cells are surrounded by a mantle of mononuclear and polymorphonuclear cells; and empty granuloma, when no parasites could be seen inside the areas of the granulomatous reaction (53). For immunohistochemical reactions, the paraffin was removed from the tissues, and antigenic recovery was performed by heating in citrate buffer (pH 6.0), for 30 min at 37°C. Endogenous peroxidase was blocked using 3% H_2_O_2_, the cells were permeabilized with 0.5% Triton, and non-specific reactions were blocked with 1% bovine serum albumin. The sections were incubated overnight with rat anti-mouse Ly6G (clone 1A8) (Biolegend, San Diego, CA, USA), or isotype control antibodies (Abcam, Cambridge, MA, USA), followed by incubation with a biotinylated secondary antibody and avidin-biotin complex (Vector Laboratories, ON, Canada). The reaction was detected with diaminobenzidine, and the sections were counterstained with Mayer’s hematoxylin. For the intracellular staining of iNOS, the liver sections were permeabilized with 0.01% saponin and incubated with rabbit anti-mouse iNOS (clone sc-649) (Santa Cruz Biotechnology, Dallas, TX, USA). Afterward, the sections were incubated with an avidin-biotin-peroxidase complex (Vector Laboratories, ON, Canada), and the color was developed using 3,3′-diaminobenzidine (Vector Laboratories). The slides were counterstained with Mayer’s hematoxylin. The areas positive for iNOS staining in the hepatic tissue were quantified using IHC Toolbox Software ImageJ (NIH, MacBiophotonics, Boston, MA, USA). The isotype control from iNOS and LY6G staining by immunohistochemistry is showed as Figure S1 in Supplementary Material.

### Evaluation of Inflammatory Infiltration in the Liver

The liver leukocytes were recovered using Ficoll-Paque PLUS gradient centrifugation. After processing, the viability was assessed using Trypan blue exclusion and the cell concentration was determined. For cytokine staining, the cells were preincubated with 20 ng/ml of PMA, 500 ng/ml of ionomycin, and Golgi Plug for 6 h; permeabilized using a Cytofix/Cytoperm kit according to the manufacturer’s instructions; and stained with α-IL-17A conjugated with Alexa700 and α-IFN-γ conjugated with APC-CY7. For FoxP3 labeling, the Foxp3 Staining Kit was used according to the manufacturer’s recommendations. For each sample, data from a minimum of 200,000 cells were acquired using a FACSCanto II flow cytometer and analyzed using FlowJo software (Tree Star, OR, USA).

### Splenic Cell Culture and Cytokine Measurement

Single-cell suspensions from the spleens of the A_2A_R^−/−^ or WT mice at various time points of infection were prepared aseptically, diluted to a concentration of 2 × 10^6^ cells/ml, and dispensed into 48-well plates in a total volume of 500 µl of complete RPMI-1640 medium (1 × 10^6^ cells/well; Gibco) with or without soluble *Leishmania* antigen (SLA) (5 µg/ml). The cell culture supernatants were harvested after 72 h of culture at 37°C in 5% CO_2_, and levels of IFN-γ and IL-10 were determined using ELISA with commercial kits (BD Biosciences and R&D Systems, Minneapolis, MN, USA).

For leukocyte identification, the inflammatory cells were gated based on their characteristic size (FSC) and granularity (SSC), and the T lymphocytes (CD4^+^ CD3^+^) and neutrophils (Ly6G^high^MHCII^−^) were individually identified. For intracellular staining, the cells were previously cultured with PMA (50 ng/ml) and ionomycin for 4 h, permeabilized with a Cytofix/Cytoperm kit (BD Biosciences) according to the manufacturer’s guide and stained with APC-Cy7-labeled α-IFN-γ or Alexa700-labeled α-IL-17 and surface-stained with FITC-labeled α-CD3 and PerCP-labeled α-CD4. For the neutrophil activation analysis, the cells were stained with antibodies to α-Ly6G, α-CD11b, α-CXCR2, and α-CD69 with APC, FITC, PERCP, and PEcy7, respectively. Foxp3 labeling were carried out using the Mouse Foxp3 Buffer Set (BD Pharmigen™) and Foxp3 (Alexa 647) antibodies. The isotype controls used were rat IgG2b and rat IgG2a. All antibodies were from BD Biosciences or eBiosciences (San Diego, CA, USA). The cell acquisition was performed using a FACSort flow cytometer. The data were plotted and analyzed using the FlowJo software (Tree Star, Ashland, OR, USA). Gating strategies were determined represented the leukocyte counts were determined by measuring the relative proportions of the leukocyte subpopulations that stained with the specific antibodies in a total of 200,000 acquired events relative to the total leukocyte numbers obtained in a Neubauer chamber. The Strategy gate for identification of inflammatory leukocytes during *L. infantum* infection was showed as Figure S2 in Supplementary Material.

### IFN-γ Depletion *In Vivo*

WT and A_2A_R^−/−^ mice were treated (i.p.) with the monoclonal antibody anti-mouse IFN-γ clone XMG1.2 (BioXCell, West Lebanon, New Hampshire, EUA). Briefly, the mice were given 20 µg of antibody 1 day before and a second dose 24 h after the infection. From the fourth week post infection, the animals were treated with 10 µg every 3 days for 2 weeks thereafter. The control group was treated with an irrelevant mab IgG following the same schedule.

### T Cell Proliferation, T Cell Isolation, and Th1 Differentiation

Spleens from naïve WT or A_2A_R^−/−^ mice and the cells were filtered through a cell strainer, centrifuged at 500 × *g* at 4°C for 10 min, and resuspended in RPMI-1640 medium at 2.5 × 10^6^ cells/ml. The cells were stained with CSFE and stimulated with plate-bound α-CD3 mAb (2 µg/ml) and α-CD28 (1 µg/ml), or medium for 4 days in a total volume of 500 µl per condition. The lymph proliferation was determined by CFSE staining by flow cytometry assay. For Th1 differentiation, CD4^+^ T cells were isolated from spleen cell suspensions of naïve WT or A_2A_R^−/−^ and stimulated with plate-bound α-CD3 (2 µg/ml), α-CD28 (1 µg/ml) (both from BioXCell) for 4 days in RPMI-1640 medium supplemented with 5% FBS (Gibco), 100 U/ml penicillin/100 µg/ml streptomycin, 1 mM sodium pyruvate, non-essential amino acids, l-glutamine and 50 µM 2-mercaptoethanol in the presence of recombinant cytokines. For Th1, differentiation was included IL-12 (5 ng/ml) plus anti-IL-4 (10 µg/ml) in addition to IL-2 (25 U/ml). All recombinant cytokines were obtained from RD and neutralizing antibodies were obtained from BioXCell. After 4 days of culture, differentiated cells were reestimulated with PMA (50 ng/ml), ionomycin (500 ng/ml) (Sigma-Aldrich), and brefeldin A for 4 h.

### Quantitative Polymerase Chain Reaction (qPCR)

The total RNA was extracted from the tissues using the TRIzol reagent (Invitrogen, Carlsbad, CA, USA) and the SV Total RNA Isolation System Kit (Promega, Madison, WI, USA) according to the manufacturer’s instructions. Complementary DNA was synthesized using Transcriptase Reverse SuperScript III (Invitrogen). SYBR Green Mix-based quantitative PCR assays were performed using the StepOnePlus Real-time PCR System (Applied Biosystems, Singapore, Malaysia). The mean threshold cycle (*C*_t_) values of duplicate measurements were used to calculate the expression of the target gene, which was normalized to the housekeeping genes *Actb, B2m* and *Gapdh* using the $2^{-\Delta \Delta C_{\text{t}}}$ formula. The standard PCR conditions were as follows: 50°C for 2 min, 95°C for 2 min and 40 cycles of 15 s at 95°C, 30 s at 58°C and 30 s at 72°C, followed by a standard denaturation curve.

### Statistical Analysis

The data are expressed as the means ± SD and are representative of two to four independent experiments. The results from individual experiments were not combined because they were analyzed individually. The means of the different groups were compared by ANOVA followed by Tukey’s honest significant difference test. Comparisons between two groups were performed using Student’s *t*-test. The analyses were performed using Prism 5.0 software (GraphPad). Statistical significance was set at *P* < 0.05.

## Results

### A_2A_R Contributes to Susceptibility during *In Vivo L. infantum* Infection

**Figure 1:**
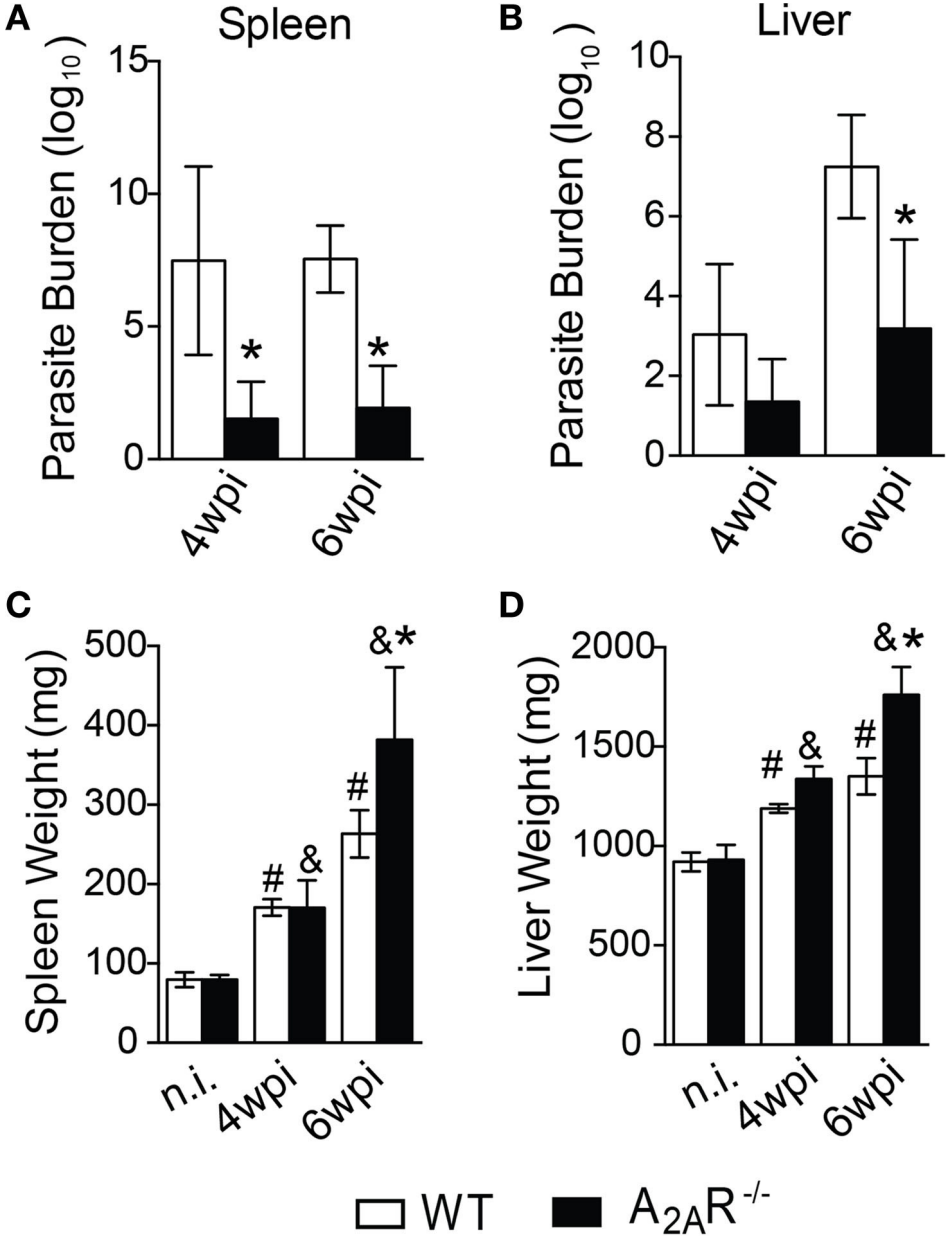
A_2A_R signaling confers the susceptibility to *Leishmania infantum* infection. BALB/c (*n* = 5) and A_2A_R^−/−^ mice (*n* = 5) (white bars and black bars, respectively) were infected with 1 × 10^7^ *L. infantum* promastigotes (HU-UFS 14) in stationary growth phase by the intravenous route (i.v.). The parasite burdens (Log10 scale) in the spleens **(A)** and livers **(B)** during the fourth and sixth weeks post infection (wpi) are shown. The spleen **(C)** and liver **(D)** weights (mg of tissue) from the non-infected (n.i.) and infected WT and A_2A_R^−/−^ groups at the fourth and sixth weeks post infection are shown. The results are expressed as the means ± SD, *P < 0.05 compared to the infected WT group, ^#^*P* < 0.05 compared to the non-infected WT group, and ^&^*P* < 0.05 compared to the uninfected A_2A_R^−/−^ group.

To investigate the relevance of A_2A_R in the course of *L. infantum* infection, A_2A_R^−/−^ mice and control littermates were intravenously infected with 10^7^ promastigote forms of *L. infantum*, and the parasite loads in the spleens and livers were quantified at various times after the infection. Strikingly, and in contrast to the WT counterparts, the spleens of the mice lacking A_2A_R harbored reduced parasite loads in both periods (Figure 1A); whereas, in the livers, fewer parasites were observed only at the sixth wpi (Figure 1B). This result suggested that A_2A_R participates in the establishment of visceral *Leishmania* infection. Surprisingly, the A_2A_R^−/−^ mice exhibited increased weights of both spleen and liver compared to the WT mice (Figures 1C,D), which may be consequence of an inflammatory reaction.

**Figure 2:**
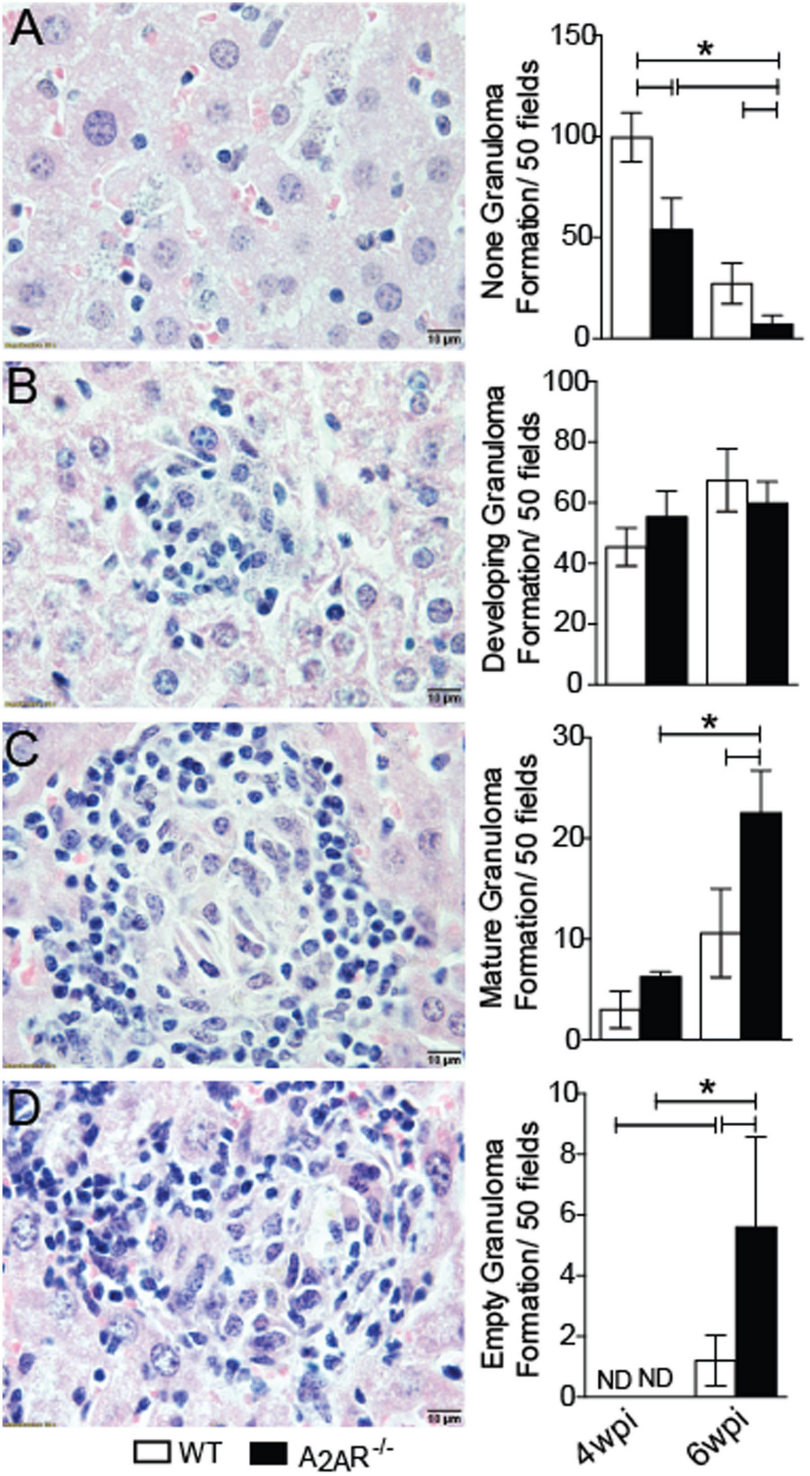
A_2A_R disturbs the granuloma reaction during *Leishmania infantum* infection. WT and A_2A_R^−/−^ mice at fourth and sixth weeks post infection were scored for the extent of granuloma formation and classified as: no granuloma **(A)**, developing granuloma **(B)**, mature granuloma **(C)** and empty granuloma **(D)**. The data are expressed as the number of granulomas per 50 high-power fields (magnification 100×). The results are expressed as the means ± SD *P < 0.05. All data are representative of at least three independent experiments.

One feature of VL is the formation of granulomas in an attempt to control the spreading of parasite (2). The granulomas can be pathologically classified as: no granuloma reaction, developing granulomas, mature granulomas, and empty granulomas (53). Histopathological analysis demonstrated that the A_2A_R^−/−^ mice exhibited larger areas with well-formed granulomas at sixth wpi than those observed in the WT mice. These areas included mature granulomas (Figure 2C) and empty granulomatous reactions (Figure 2D). These results were consistent with the lower parasite numbers detected in the livers (Figure 1B) and dearth areas with none granuloma reaction (Figure 2A). Moreover, we could not detect any difference with respect to the developing granuloma reactions during the fourth and sixth wpi between the WT and A_2A_R^−/−^ mice (Figure 2B). These data demonstrated that activation of A_2A_R results in the failure of BALB/c mice to control the *L. infantum* infection possibly due to the generation of a weaker cellular immune response.

### A_2A_R Inhibits the Th1 Response during *L. infantum* Infection

**Figure 3:**
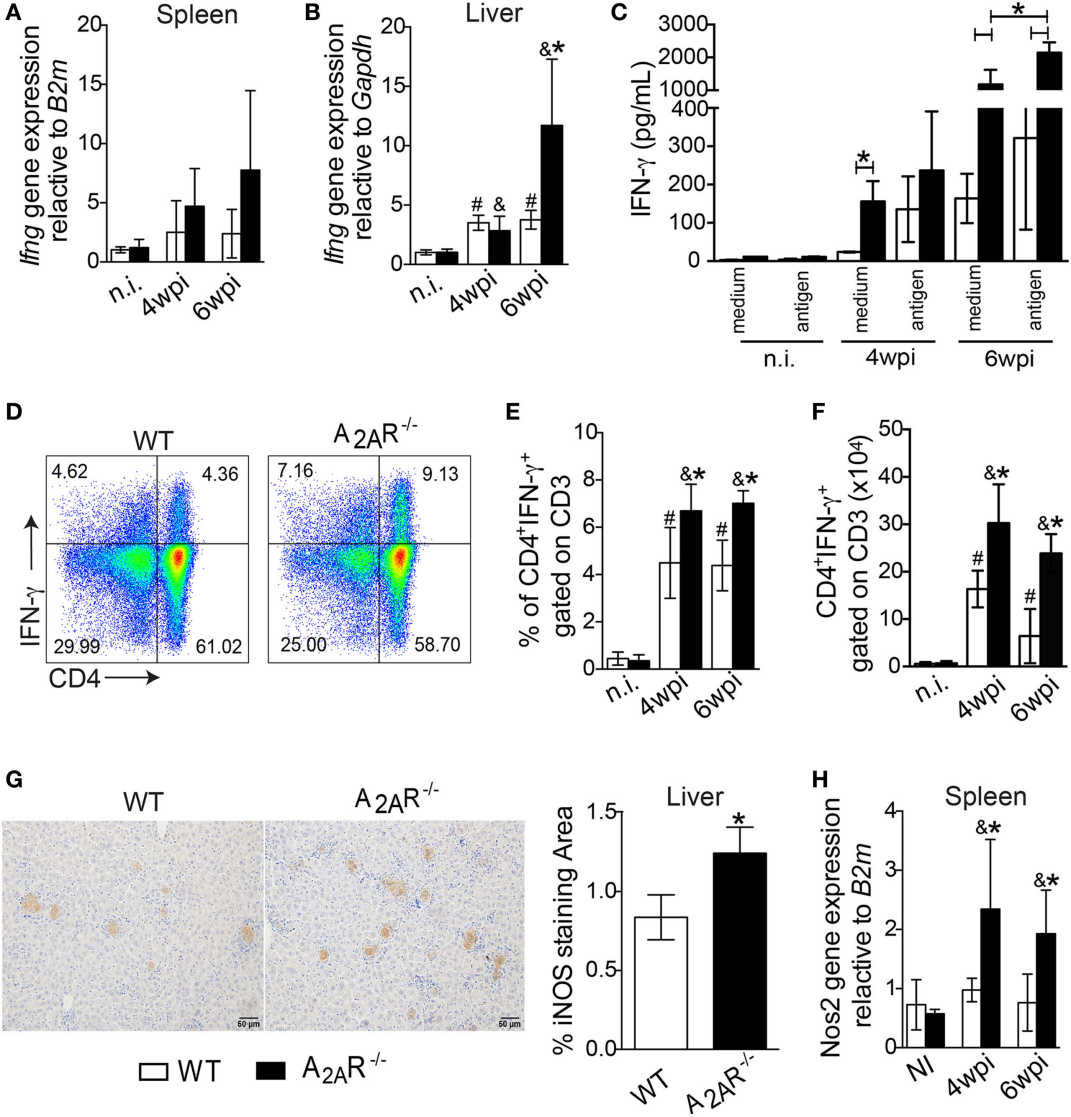
A_2A_R regulates the establishment of the Th1 immune response in the course of *Leishmania infantum* infection. Fragments from the spleens **(A)** and livers **(B)** from the WT (white bars, *n* = 5) and A_2A_R^−/−^ (black bars, *n* = 5) mice were subjected to quantitative polymerase chain reaction (qPCR) analysis to evaluate the expression of *ifng* mRNA. The IFN-γ produced by the splenic cells of the non-infected and infected WT and A_2A_R^−/−^ mice in the presence or absence of *L. infantum* antigens is shown **(C)**. The results are expressed as the means ± SD, *P < 0.05 for the comparison with *Leishmania* antigen or medium stimuli. The dot plots represent the frequency of CD4^+^ IFN-γ^+^ gated on CD3^+^ cells **(D)**. The frequencies **(E)** and absolute numbers **(F)** of CD4^+^ IFN-γ^+^ gated on CD3^+^ cells that were present in the spleens of the non-infected (n.i.) and infected mice at fourth and sixth weeks of infection are shown. Representative photos of the NOS2 staining by immunohistochemistry in the hepatic tissues from the WT (*n* = 5) and A_2A_R^−/−^ (*n* = 5) mice at the sixth wpi **(G)**. The photomicrographs are shown at 20X magnification. The positive percentage of NOS2-stained area at the sixth wpi was quantified using ImageJ^®^ software analysis. The mRNA for *nos2* expression in spleen was quantified using qPCR **(H)**. The results are expressed as the means ± SD, ^#^*P* < 0.05 compared to the non-infected WT, ^&^*P* < 0.05 compared to the non-infected A_2A_R group, and *P < 0.05 for the comparison of the infected WT group.

Because the development of IFN-γ-producing CD4^+^ T helper cells is crucial for the control of parasite replication in the target organs of VL, we investigated whether this response could be affected in A_2A_R-dependent manner. The *Ifng* gene expression was upregulated in the livers (Figure 3B), but it not altered into spleen (Figure 3A), of the A_2A_R^−/−^ mice at the sixth wpi compared to the control littermates. In terms of protein, IFN-γ production was measured in response to restimulation of the spleen cells from A_2A_R^−/−^ or WT mice with the SLA. Stimulation of the cells from the WT mice with SLA induced the release of significant amounts of IFN-γ into culture supernatant compared with WT control (medium-without SLA) Figure 3C. However, the supernatants of the spleen cells from the A_2A_R^−/−^ mice contained higher levels of this cytokine by in comparison with supernatants stimulated WT cells. This profile was weakly observed at the fourth wpi, but it was pronounced at the sixth wpi. Moreover, there is a significant difference in the amounts of IFN-γ into culture supernatants from antigen-stimulated A_2A_R^−/−^ compared with respective A_2A_R^−/−^ control (medium- without SLA). The *Leishmania* antigen had minimal effects on the basal IFN-γ release in either strain of non-infected mice.

By flow cytometry, we observed that infection with *L. infantum* promoted a significant induction of IFN-γ-producing CD4^+^ T cells in both BALB/c and A_2A_R^−/−^ mice compared to their counterparts in terms of both percentages (Figures 3D,E) and total number of cells (Figure 3F). However, it must be noted that the previously mentioned Th1 profile enhancement was heightened in A_2A_R^−/−^ mice. Moreover, the infection promoted the expansion of the CD4^+^ IL-17^+^ gated on CD3^+^ cells, but such population was not affected in the absence of the receptor. There were no differences between the WT and A_2A_R^−/−^ mice in the frequencies and absolute numbers of the CD4^+^ IL-17^+^ gated on CD3^+^ (Figures S3A,B in Supplementary Material). We also could not see any difference in the frequency and number of IL-17 production by other CD3^+^ population, herein characterized as CD4^−^ IL-17^+^ gated on CD3^+^, that could be CD8^+^ T, NK^+^ T, or γδ^+^ T cells between WT and A_2A_R^−/−^ (Figures S3A,C in Supplementary Material).

A considerable body of evidence has shown that the Th1 subset produces IFN-γ, which in turn induces expression of iNOS (NOS2) in infected phagocytes, which generates NO (54). The resulting production of NO by iNOS represents an important tool to kill *Leishmania* sp parasites (5, 6). By qPCR, we detected greater *Nos2* mRNA expression in the spleens of the A_2A_R^−/−^ mice than in the WT group at both the fourth and sixth weeks post infection (Figure 3H). Likewise, greater areas positive for iNOS staining were observed in the livers of the knockout mice at sixth week post infection than in the WT group (Figure 3G). These results suggested that A_2A_R activation downregulated the Th1 responses which could favor the parasite spreading.

### A_2A_R Regulates both Neutrophil Recruitment and Activation through an IFN-γ-Dependent Mechanism

Neutrophils are recruited to *Leishmania* inoculation foci (55) and participate in the restriction of the parasites during VL (56, 57). Because neutrophils both produce and respond to adenosine (58), we addressed whether A_2A_R signaling affected the neutrophilic inflammation in target organs of the disease.

**Figure 4:**
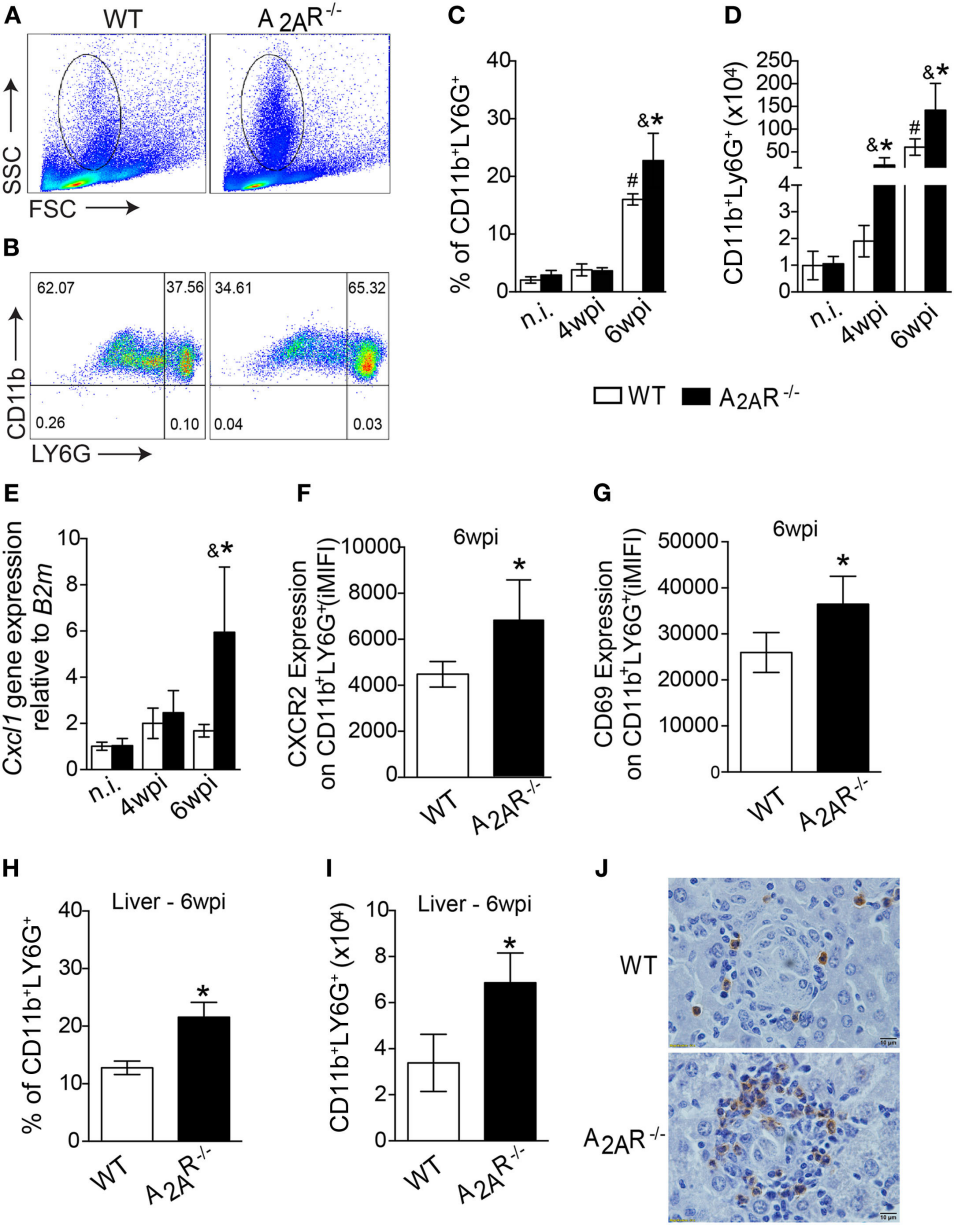
A_2A_R decreases the neutrophil recruitment to the foci of infections during *Leishmania infantum* infection. Granulocytes were identified according to their size (FSC) and granularity (SSC) **(A)** and further neutrophils were characterized as CD11b^+^ Ly6G^+^ cells **(B)** by flow cytometric analysis. The dot-plots represent the frequency **(C)** and the absolute number **(D)** of CD11b^+^ LY6G^+^ cells in the spleens of the non-infected (n.i.) and infected (4 wpi and 6 wpi) WT (white bars, *n* = 5) and A_2A_R^−/−^ (black bars, *n* = 5) mice. *Cxcl1* gene expression in the spleens from non-infected (n.i.) and infected WT and A_2A_R^−/−^ mice (4 wpi and 6 wpi) **(E)**. Expression of CXCR2 **(F)** and CD69 **(G)** (integrated median fluorescence intensity) on surface of CD11b^+^ LY6G^+^ cells at 6 wpi in the spleens of the WT (white bars, *n* = 5) and A_2A_R^−/−^ (black bars, *n* = 5) animals. The percentages **(H)** and absolute numbers **(I)** of CD11b^+^ LY6G^+^ cells in the livers of WT and A_2A_R^−/−^ mice at sixth wpi determined by flow cytometry are shown. Representative photos of Ly6G staining by immunohistochemistry in the hepatic tissue at the sixth wpi **(J)** are shown. The photomicrographs are shown at 100X magnification. The results are expressed as the means ± SD, ^#^*P* < 0.05 compared to the non-infected WT group, ^&^*P* < 0.05 compared to the non-infected A_2A_R^−/−^ group, and *P < 0.05 compared to the infected WT group.

On the basis of the size (FSC) and granularity (SSC) characteristics, we found a higher frequency of leukocytes with a high side-scatter height, a classical gate for granulocytes, in the infected A_2A_R^−/−^ mice compared to the infected WT mice (Figure 4A). Using specific antibodies to identify the neutrophils, we observed differences in absolute numbers of Ly6G^+^ CD11b^+^ cells (neutrophils markers) at fourth wpi and the most notable difference was that the neutrophilic influx observed at sixth wpi that was greater both in percentage (Figures 4B,C) and total number (Figure 4D) of cells in the infected A_2A_R^−/−^ mice than in the littermate controls. Interestingly, the strong neutrophilic influx in the absence of A_2A_R was associated with the increased expression of *cxcl1*, which codes for an important neutrophil chemotactic mediator, by the spleen cells (Figure 4E). Subsequently, we used flow cytometry to examine the surface molecules expressed on surface of CD11b^+^ Ly6G^+^ cells to determine whether differences in expression of CXCR2 or CD69, relative to neutrophil migration (59–61) and activation (62, 63), respectively, could account for the strong inflammatory process encountered in the tissues of the infected A_2A_R^−/−^ mice. Consistent with these observations, the neutrophils from the A_2A_R^−/−^ mice exhibited an enhanced expression of CXCR2, the CXCL1 receptor (Figure 4F; Figure S4 in Supplementary Material). Among the neutrophil activation marker, we observed that the integrated median fluorescence intensity (iMFI) for CD69 from the A_2A_R^−/−^ neutrophils was significantly higher than the iMFI for these markers on the WT neutrophils (Figure 4G; Figure S4 in Supplementary Material). Likewise, consistent with the spleen results, the liver sections from the A_2A_R^−/−^ mice showed a marked increase in the stained neutrophils that infiltrated into the hepatic granulomas (Figure 4J). By flow cytometry, higher neutrophils were detected into liver of A_2A_R^−/−^ mice (Figures 4H,I), which suggests that both neutrophil migration and activation may be affected by the adenosine receptor during VL.

**Figure 5:**
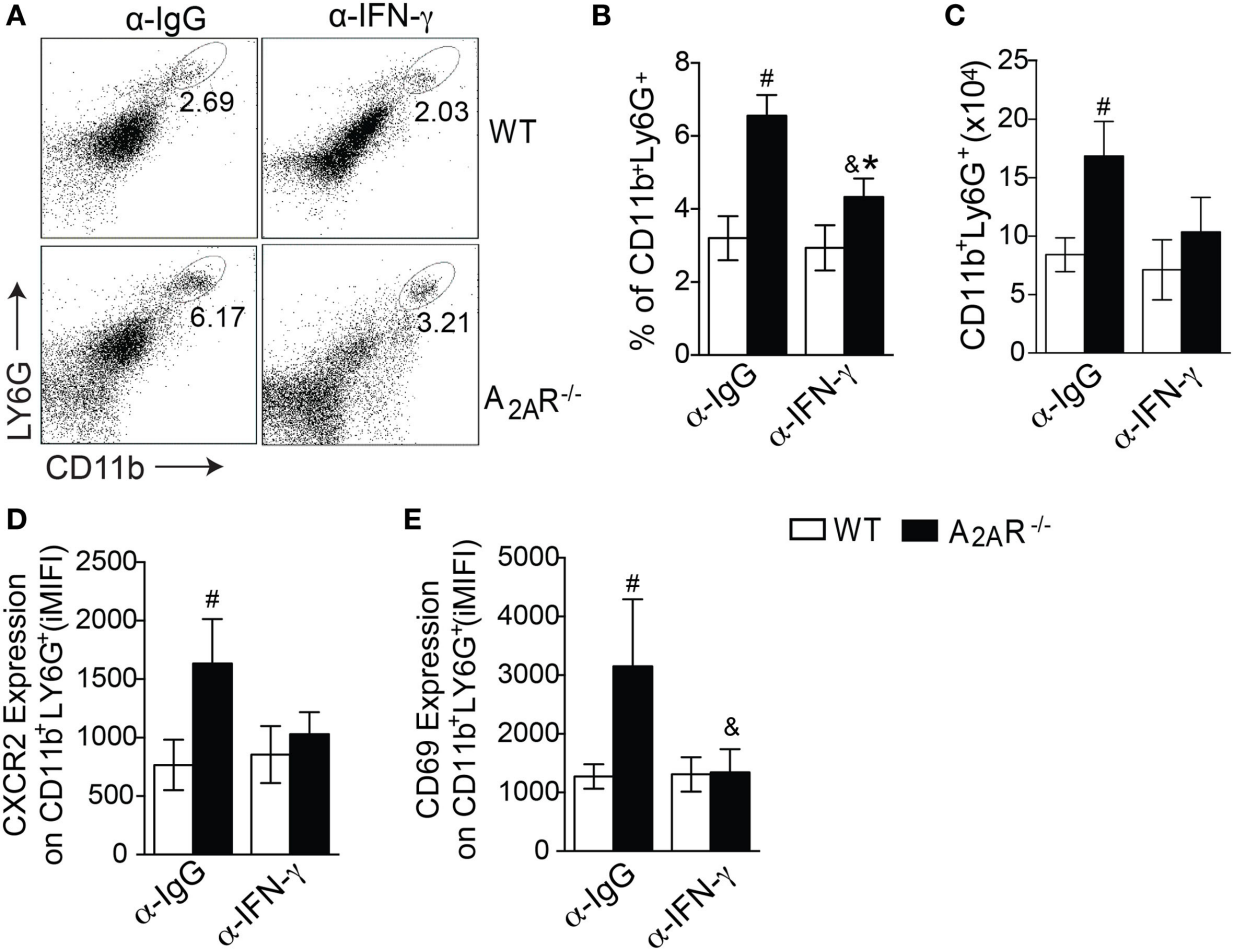
A_2A_R regulates the neutrophil activation and recruitment into the spleen in an IFN-γ-dependent fashion. The dot plots represent the frequency of CD11b^+^ Ly6G^+^ cells gated on granulocytes **(A)**. The frequencies **(B)** and absolute numbers **(C)** of splenic CD11b^+^ Ly6G^+^ cells at the sixth wpi in the WT (white bars) and A_2A_R^−/−^ (black bars) mice that were treated with a control antibody (α-IgG) or interferon-γ neutralizing antibodies (α-IFNγ) (*n* = 6 mice per group). The medians of the integrated median fluorescence intensity of CXCR2 **(D)** and CD69 **(E)** gated on CD11b^+^ Ly6G^+^ cells from the non-treated infected mice or those treated with α-IFNγ at the sixth wpi are shown. The results are expressed as the means ± SD, ^#^*P* < 0.05 compared to the α-IgG-treated WT mice, ^&^*P* < 0.05 compared to the α-IgG-treated A_2A_R^−/−^ mice, and *P < 0.05 compared to the α-IFN-γ-treated WT group.

It has been determined that IFN-γ directly modulates neutrophil behavior to favor the expression of molecules involved with cell adhesion, migration, activation, and killing activity (64). To assess whether IFN-γ could be responsible for the neutrophilic influx into the target organs of the A_2A_R^−/−^ mice, we blocked IFN-γ using a specific antibody in the infected mice. The treatment with the α-IFN-γ antibody abrogated the neutrophilic inflammation, as observed by the reduction of percentage (Figures 5A,B) and total number (Figure 5C) of CD11b^+^ LY6G^+^ cells in the spleens of the A_2A_R^−/−^ mice. In addition, a smaller surface expression of CXCR2 (Figure 5D) and CD69 (Figure 5E) was observed in the A_2A_R^−/−^ mice treated with α-IFN-γ than with respective group treated with α-IgG. Interestingly, none of these parameters differed between the WT mice that were treated with α-IFN-γ or anti-IgG control. Together, these data suggested that A_2A_R may modulate the Th1 subset that is capable of inducing neutrophilic inflammation and controlling the parasite spreading into tissue.

### The Immunoregulatory Effect of A_2A_R Pathway Is Related to the Tregs and IL-10 Production

To determine the mechanism by which A_2A_R modulates Th1 responses during *L. infantum* infection, we first examined whether T cell-intrinsic A_2A_R is involved in either T cells proliferation or Th1 generation. CFSE-labeled CD4^+^ T cells isolated from the spleens of naïve WT or A_2A_R^−/−^ mice cultured under Th0 and Th1 condition were stimulated with α-CD3 + α-CD28 for 4 days. The proliferation assay was analyzed by CFSE-positivity and Th1 differentiation by either intracellular stained-IFN-γ production or T-bet mRNA expression. The frequency of CFSE-labeled CD4^+^ T cells between A_2A_R^−/−^ in the presence of polyclonal stimuli was similar to that in WT cells (Figures S5A,B in Supplementary Material). Moreover, the IFN-γ-production by A_2A_R^−/−^ CD4^+^ T cells maintained under Th1 condition were induced in similar levels that those in WT cells (Figure S5C in Supplementary Material). There was no significant difference in T-bet mRNA expression by WT and A_2A_R^−/−^ CD4^+^ T cells under Th1 polarizing condition (Figure S5D in Supplementary Material), indicating that T cell-intrinsic A_2A_R does not affect the either proliferation or differentiation of Th1 cells.

**Figure 6:**
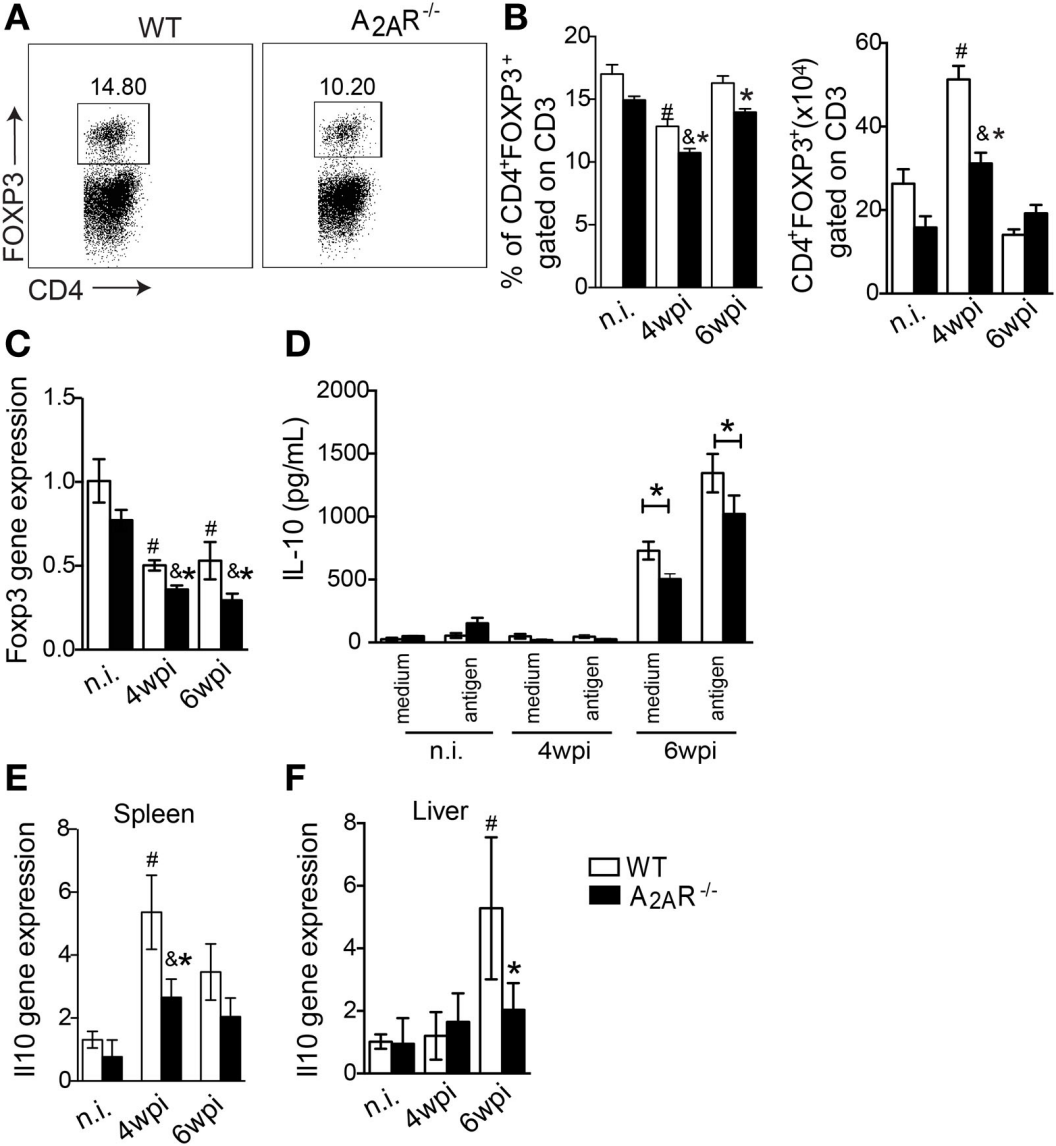
A_2A_R induces the regulatory T cell population and IL-10 production during *Leishmania infantum* infection. In **(A)** representative dot plots of CD4^+^ FOXP3^+^ gated on CD3^+^ cells in spleen of infected WT or A_2A_R^−/−^ are shown in each box. The frequencies and absolute numbers of splenic CD4^+^ FOXP3^+^ gated on CD3^+^ cells in non-infected (n.i) and infected WT and A_2A_R^−/−^ mice (white bars and black bars, respectively) at the fourth and sixth weeks post infection **(B)**. The levels of the mRNA for *foxp3* expression in the livers were determined by quantitative polymerase chain reaction **(C)**. The levels of IL-10 produced by the splenic cells cultured at different phases of infection in the presence or absence of *L. infantum* antigens are shown **(D)**. The *il10* expression in spleens **(E)** and livers **(F)** of the infected and non-infected groups is shown. The results are expressed as the means ± SD. ^#^*P* < 0.05 compared to the uninfected WT group, ^&^*P* < 0.05 compared to the non-infected A_2A_R^−/−^ group, and *P < 0.05 compared to the infected WT group.

Taking into account that A_2A_R signaling can restore homeostasis by promoting Tregs generation and immunosupression (27) and inflammatory mediators such as IFN-γ can limit the Treg function and differentiation (65) and that IFN-γ was upregulated in the infectious foci of *L. infantum* (Figures 3A–F), we next evaluated whether the Th1-induced inflammation observed in the A_2A_R^−/−^ mice could be related to compromised Treg functions during infection. The results showed that following the infection, the frequency of CD4^+^ T Foxp3^+^ cells were reduced in both infected groups at fourth wpi compared with respective naïve littermate control group. However, the reduction of CD4^+^ T Foxp3^+^ cells was more pronounced on A_2A_R^−/−^ mice when compared to infected WT mice (Figures 6A,B). In terms of total cells, despite the infection promoting CD4^+^ T Foxp3^+^ cells expansion on both infected groups at fourth wpi, it was reduced on infected A_2A_R^−/−^ mice when compared to infected WT compared with respective non-infected littermate controls (Figures 6A,B). Moreover, *foxp3* mRNA expression was reduced in the livers of A_2A_R^−/−^ mice (Figure 6C). We previously demonstrated that adenosine provided an anti-inflammatory activity through a mechanism that was dependent on PGE_2_-induced IL-10 release (29). Consistent with the observed Treg reduction, the transcripts of IL-10, an important anti-inflammatory cytokine that is released through A_2A_R signaling, was reduced in the spleens at fourth wpi (Figure 6E) and livers at sixth wpi (Figure 6F) of the knockout mice. We observed a similar inhibition in the IL-10 release into supernatants of the *L. infantum* antigen-stimulated spleen leukocytes from the A_2A_R^−/−^ mice compared with the stimulated cells from the infected WT mice (Figure 6D).

## Discussion

This study revealed the role of A_2A_R in increasing the susceptibility to *L. infantum* infection during experimental VL. Herein, we demonstrated that the absence of A_2A_R in the susceptible lineage BALB/c mice increased the cellular immune reaction as shown histopathologically by raised increased areas of mature and empty granulomas into liver. To understand the role of A_2A_R signaling, we quantified lower parasite burden in the A_2A_R^−/−^ mice that was accompanied by stronger development of the Th1 pattern of immune response, as indicated by a higher frequency of IFN-γ-producing T cells, increased iNOS expression in the spleens and livers, and decreased Treg numbers and IL-10 release.

Several lines of evidence have shown that the development of mature hepatic granulomas is strictly related to the parasite killing, in that it limits spreading of the parasites to the organs (66). Accordingly, the A_2A_R^−/−^ mice at the sixth wpi harbored fewer parasites in the livers, and this was related to the elevated numbers of mature granulomas and empty granulomas in the hepatic tissue. Conversely, the susceptibility of WT mice during *L. infantum* infection was accompanied by a higher frequency of no granuloma reactions in both analyzed periods. It is possible that this was due to a decreased capacity to generate the mature granulomas that limit the parasite spreading.

A major requirement for maturation of a granuloma is the IFN-γ production (2, 3). In this sense, the elevated ratio of mature granulomas reflected the increased cellular immune reaction in the A_2A_R^−/−^ mice, which presented a strong Th1 adaptive immune reaction during *L. infantum* infection. It is no surprise that during a *Leishmania* infection, IFN-γ causes activation of the phagocytes and production of NO, a molecule that is importantly involved in parasite killing (5, 6). However, it is important to note that the increased frequency of mature granulomas was directly accompanied by increased areas of NOS2 staining in the liver, which reflects the importance of IFN-γ in limiting the hepatic infection.

It has been demonstrated that A_2A_R is upregulated after T lymphocyte activation (25) and attenuates T cell response against cognate stimuli (27). Accordingly, the induction of experimental VL in A_2A_R^−/−^ mice resulted in an increased Th1 cell frequency during the infection as well as an attenuated response of the splenocytes to *ex vivo* stimulation with *L. infantum* antigen as demonstrated by lower levels of IFN-γ production. Thus, these data could explain the reduced areas of the no granuloma reactions and the increased areas of both mature and empty granulomas on A_2A_R^−/−^ mice, which reflected the high expression of iNOS into target organs.

The neutrophil recruitment to the VL-infected organs is crucial to controlling the parasite replication (67). Our data demonstrated that the infected A_2A_R^−/−^ mice displayed an enhanced migration of activated neutrophils into the infected foci, a phenomenon that was accompanied by an increased capacity to control the spreading of the parasites into target organs. It has been shown that Th1 pattern-derived cytokines upregulate A_2A_R on phagocytes (68). This receptor is mainly involved with inhibition of cell migration, ROS production, and phagocytic activity (20, 22, 69). Interestingly, the absence of A_2A_R upregulated the expression of the neutrophil chemoattractant *Cxcl1* and its receptor CXCR2 on neutrophils, all of which are events that are involved in neutrophil recruitment. Regarding the activation, CD69 was highly expressed on neutrophils from infected A_2A_R^−/−^ mice, a phenomenon that could be related to the IFN-γ release (62, 63). Curiously, not only was neutrophil activation abrogated after IFN-γ depletion but also their recruitment was affected in the infected A_2A_R^−/−^ mice, and this was accompanied by decreased CXCR2 expression. This result demonstrated that the A_2A_R-mediated regulation of neutrophil recruitment during the *L. infantum* infection resulted from the ability of this receptor to attenuate the Th1 immune response.

In addition to repressing the development of the Th1 immune response, A_2A_R signaling closely associated with the generation of Tregs and the improvement of their suppressor activity by stabilizing FOXP3 expression and inducing the CTLA-4-mediated suppressive effects (28). It is well established in the literature that suppression by the Tregs culminates in susceptibility to infection in several experimental models, including *Leishmania* (70). Accordingly, we demonstrated that the A_2A_R^−/−^ mice had a reduced frequency of Tregs during infection. It is also known that the Tregs represent a relevant source of adenosine through the action of the ectonucleotidases CD39 and CD73 (71). In addition to expanding the regulatory T cell repertoire, several lines of evidence highlight the importance of A_2A_R in the context of immune regulation in which it induces release of IL-10 by several types of leukocytes, including DCs, T cells, and Tregs (12, 18, 19, 72, 73). According to the literature, the lower levels of Tregs in A_2A_R^−/−^ mice are accompanied by lower levels of *il10* mRNA in the organs targeted by the disease. Moreover, the splenocytes of these mice exhibited reduced IL-10 production *ex vivo* following stimulation with the parasite, which suggested that these mice presented an attenuated immunosuppressive potential during experimental VL (72, 74, 75). IL-10 is a potent anti-inflammatory cytokine that is strictly involved with VL progression by impairing the Th1 cell responses, which abrogates the microbicidal mechanisms of the parasitized macrophages (76). Taking these data together, we hypothesize that the A_2A_R-mediated susceptibility in BALB/c mice is based in expanding the numbers of Tregs that, in turn, generate elevated levels of adenosine. This nucleoside may act in a positive feedback loop in which IL-10 is generated by Tregs in an autocrine fashion as well as in paracrine fashion by effector T cells. Moreover, we cannot exclude a role for DCs affecting the Th1 cell polarization based on the recent observation by our group that adenosine, acting through A_2A_R, modulates DC activation as well as the T cell polarization toward to an anti-inflammatory phenotype (46). However, the implications of this process were not aimed in this work. We discard an intrinsic defect of A_2A_R signaling on Th1 driving, since CD4^+^ T cells isolated from naïve A_2A_R^−/−^ under Th1 condition presented similar capacity to express T-bet transcriptional factor and IFN-γ production than those of WT cells.

An important clinical outcome during VL is the development of hepatosplenomegaly (3). The evaluation of the weights of these target organs during the experimental infection revealed that the spleens and livers of the A_2A_R^−/−^ mice were enlarged compared to the WT counterparts. Thus, we suggest that this phenomenon could be a result of the strong inflammatory response that favored the elimination of the parasites. In agreement with this result, our group observed that *Il17ra^−/−^* mice, which were more susceptible to infection by *L. infantum*, exhibited less hepatosplenomegaly than the resistant WT group (7). Interestingly, CD4^+^ T cells-producing IL-17 is also clinically associated with chronic inflammation seen in VL. Symptomatic patients showed a positive correlation between IL-17 and aspartate transaminase levels, indicating development of liver injury in those individuals (77). Therefore, the excessive inflammation triggered during VL could promote tissue damage (herein exemplified by organ enlargement) even though controlling parasite replication. Thus, we suggested adenosine signaling through A_2A_R limited the inflammation, controlling hepatosplenomegaly but it promotes parasite spread.

We conclude that A_2A_R is a negative regulator of the Th1 immune response, which may be due to an anti-inflammatory activity mediated by IL-10. In the absence of A_2A_R, BALB/c became resistant to *L. infantum* infection through an exacerbated Th1 immune response, which was responsible for recruiting neutrophils into the foci of the infection. Furthermore, we detected reduced regulatory T cell numbers and IL-10 production in the absence of A_2A_R signaling. Thus, we suggest that inhibition or blockade of A_2A_R could enhance the immune system effector functions to address persistent infections.

## Ethics Statement

All experiments were conducted in accordance with the National Institutes of Health (NIH) guidelines on the welfare of experimental animals and with the approval of the Ethics Committee of the School of Medicine of Ribeirão Preto (No 196/2011).

## Author Contributions

Conceived and designed the experiments: ML and VC. Performed the experiments: ML, LS, GQ, MF, AS, LB, and VC. Analyzed the data: ML and VC. Contributed reagents/materials/analysis tools: LB, FC, RA, JS, and VC. Wrote the paper: ML, GQ, and VC.

## Conflict of Interest Statement

The authors declare that the research was conducted in the absence of any commercial or financial relationships that could be construed as potential conflicts of interest.
