# Supplementary material for: Leishmania infantum Parasites Subvert the Host Inflammatory Response through the Adenosine A2A Receptor to Promote the Establishment of Infection
Source: Front Immunol. 2017 Jul 20;8:815. doi: 10.3389/fimmu.2017.00815 (PMC5517451; doi:10.3389/fimmu.2017.00815)
Supplement: Supplementary file 1 [file Data_Sheet_1.DOC]

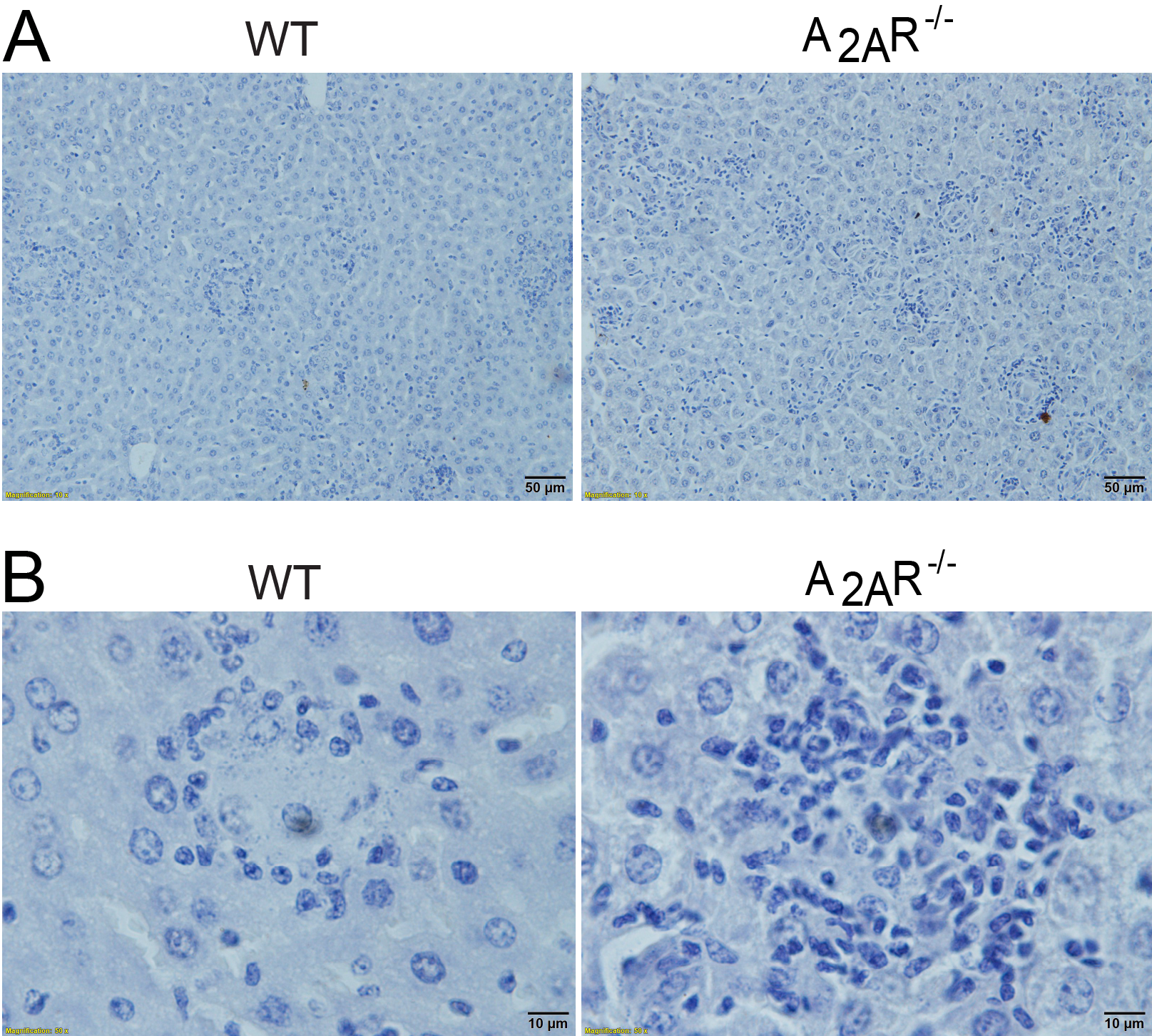


**Supplementary Figure 1-** **Isotype control from iNOS and LY6G staining by imunohistochemistry.** Representative photos of the isotype control from iNOS (A) or LY6G (B) staining by immunohistochemistry in the hepatic tissues from the WT (n=5) and A_2A_R^-/-^ (n=5) mice at the 6^th^ wpi. The photomicrographs are shown in the scale of 50 µm by iNOS isotype control and 10 µm scale by LY6G isotype control.


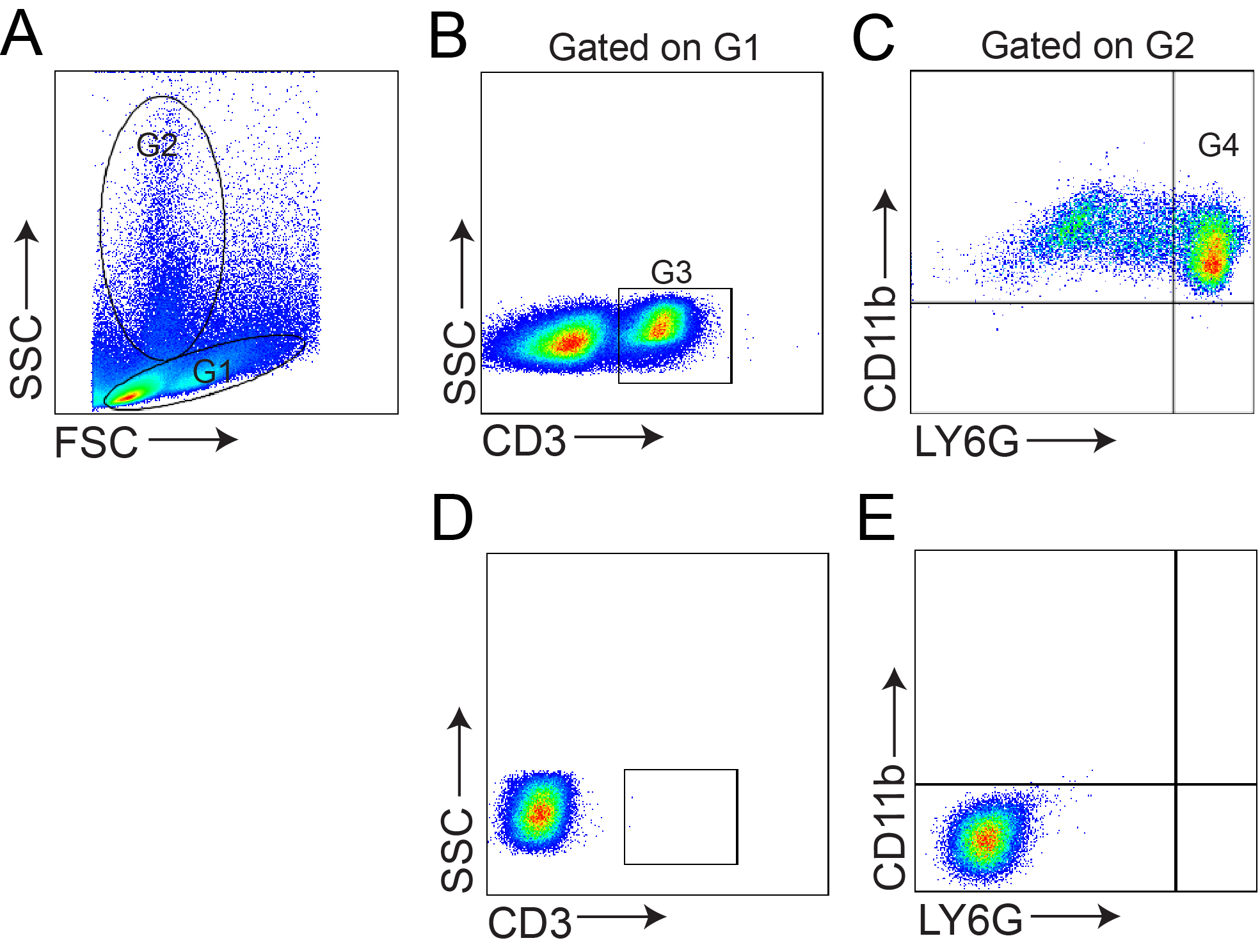


**Supplementary Figure 2 -** **Strategy gate for identification of inflammatory leucocytes during *L. infantum* infection.** For the leukocyte identification, the inflammatory cells were firstly gated based on their characteristic size (FSC) and granularity (SSC) (panel A). As gate strategy for analyzing intracellular staining by lymphocytes, CD3^+^ cells were gated on G1 (lymphocyte gate) (panel A) and CD4^+^T cells- producing IFN-γ subsets or CD4^+^T Foxp3^+^ were determined on G3 gate (panel B). For neutrophils analyses, the CD11b^+^LY6G^+^ cells were gated on G2 (panel A) and subsequent activation markers (CXCR2 and CD69) were identified individually under G4 (panel C). The respective isotype controls used for lymphocytes (panel D) or granulocytes (panel E) characterization are demonstrated.


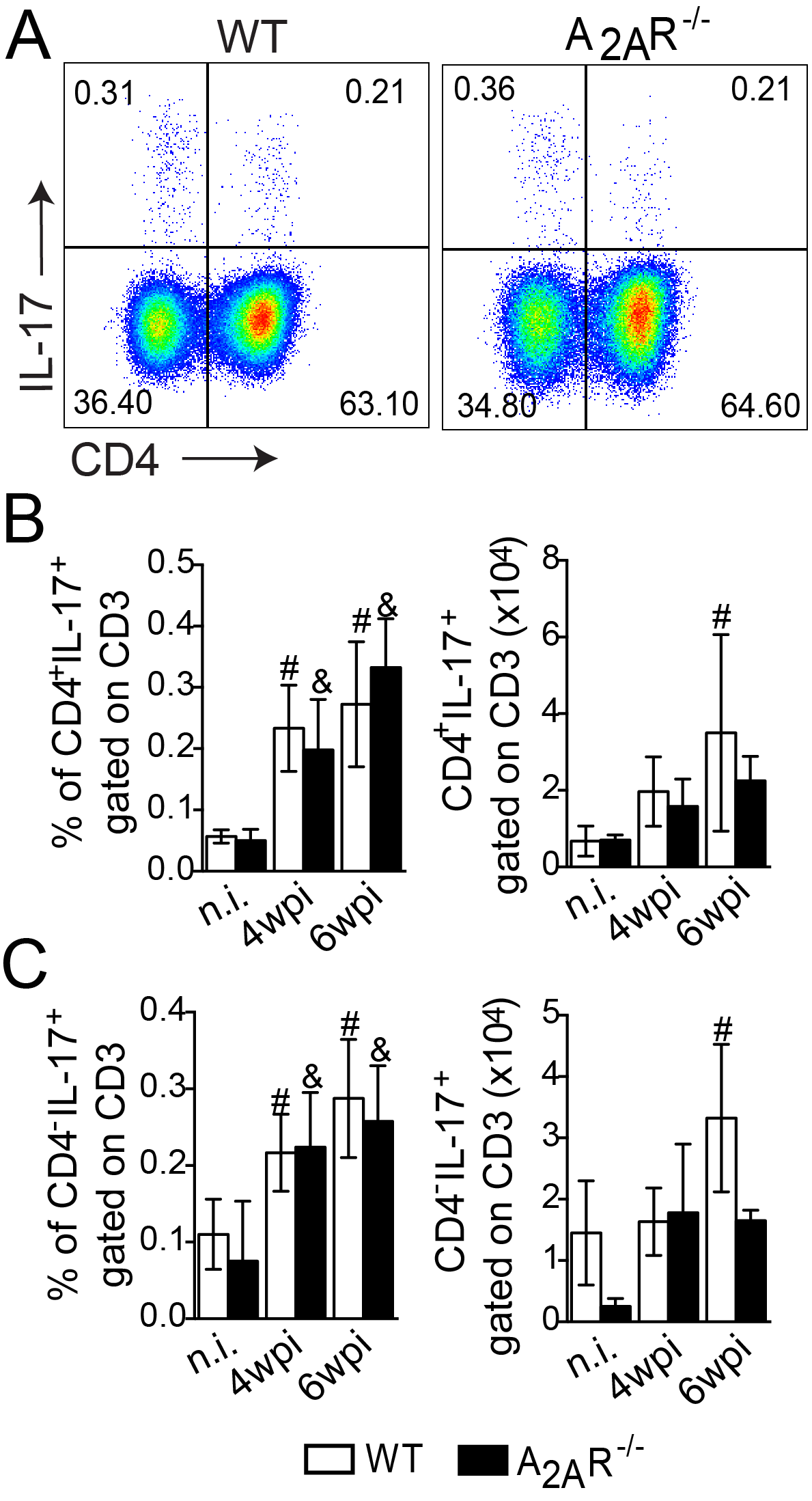


**Supplementary Figure 3- A_2A_R does not alter the production of IL-17 by distinct T lymphocytes subset during *Leishmania infantum* infection.** The lymphocytes were selected according to their size (FSC) and granularity (SSC) and further characterized as CD4^+^IL-17^+^ or CD4^-^IL-17^+^ cells gated on CD3^+^ cells by flow cytometric analysis. In A, The frequencies and absolute numbers of the splenic CD4^+^ IL-17^+^ T cells or CD4^-^IL-17^+^ T cells of the WT and A_2A_R^-/-^ mice (white and black bars, respectively) are shown. The results are expressed as the means ± SEM. ^#^*P* < 0.05 compared to the uninfected WT group, ^&^*P* < 0.05 compared to the non-infected A_2A_R^-/-^ group.^*^*P* < 0.05 compared to the infected WT group.


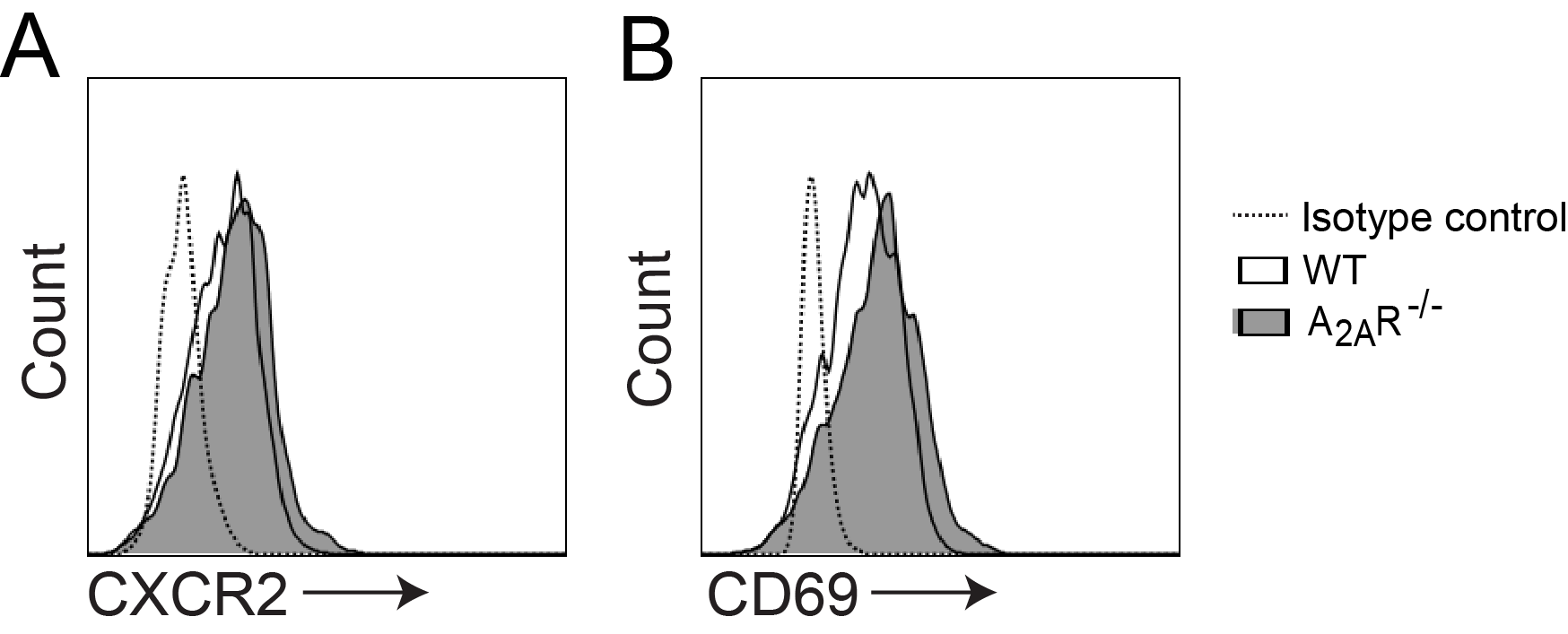


**Supplementary Figure 4- A_2A_R inhibits neutrophils activation markers.** The histograms represented the expression of CXCR2 (A) and CD69 (B) on surface of CD11b^+^LY6G^+^ cells at 6 wpi in the spleens of the WT (white bars, n=5) and A_2A_R^-/-^ (black bars, n=5) animals. The filled histograms represent cells labeled with the specific mAb; the dashed histograms represent the same cell suspension labeled with isotypic control mAb.

**
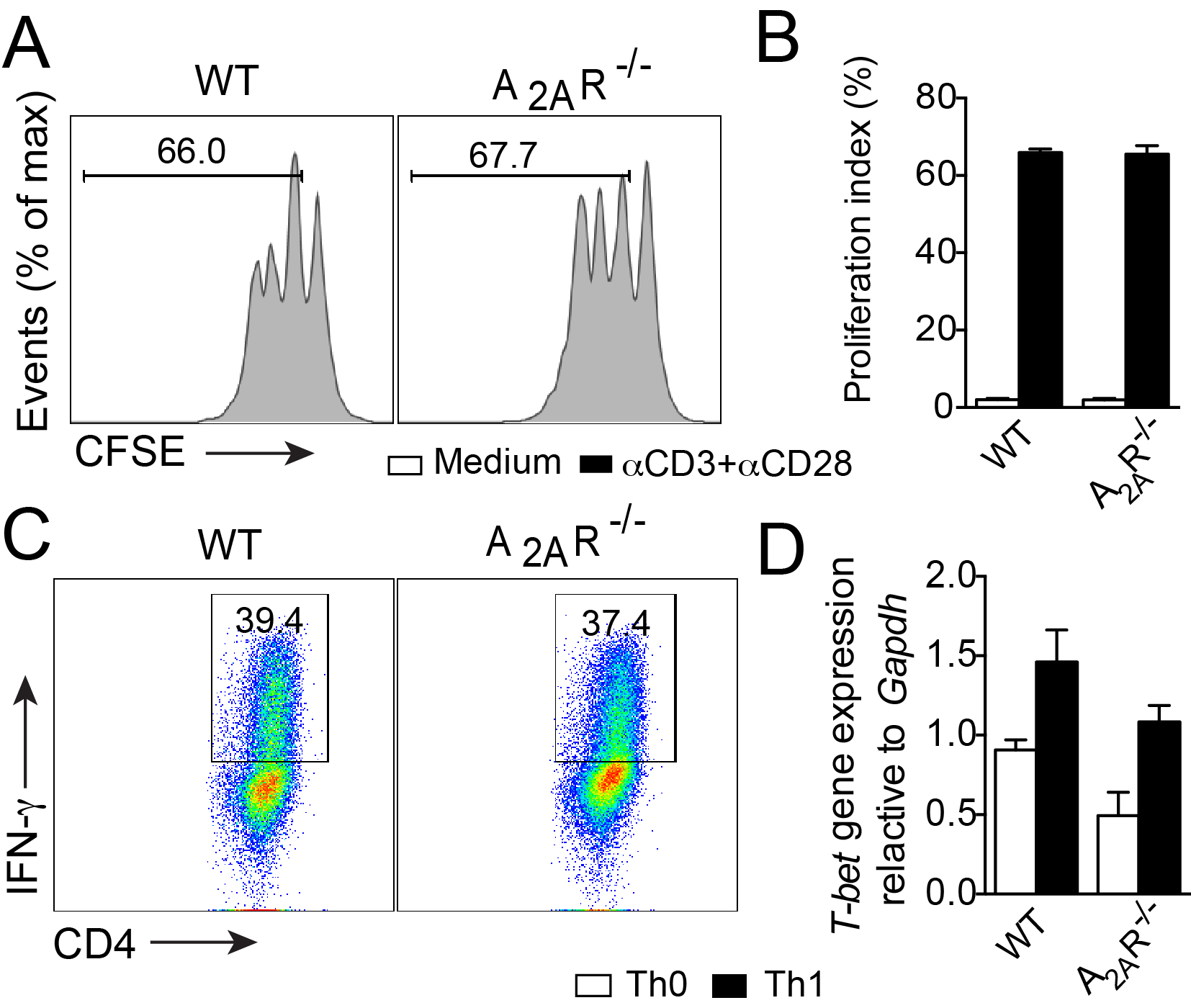
**

**Supplementary Figure 5- Normal lymph proliferation and Th1 cell differentiation of A_2A_R^-/-^- CD4^+^T cells.** Total spleen cells from naive wild-type and A_2A_R^-/-^ mice were stained with CFSE and stimulated with α-CD3 and α-CD28 or medium for 4d. Histograms reveal the proliferation of spleen cell by CFSE expression (A) and graph bars represent the media of proliferation index (B)**.** Isolated CD4^+^T cells from naïve wild-type and A_2A_R^-/-^ were cultured under Th0 or Th1 conditions for 4 d. Dot-plots represent the frequency of IFN-γ- producing CD4^+^T cells (C) and T-bet mRNA expression by RT-PCR (D). The results are expressed as the mean ± SEM obtained from quadruplicate samples from one of three independent experiments (*n* = 4 per group).
